# The Effect of Water Supply on Sweet Cherry Phytochemicals in Bud, Leaf and Fruit

**DOI:** 10.3390/plants10061131

**Published:** 2021-06-02

**Authors:** Matej Vosnjak, Davor Mrzlic, Metka Hudina, Valentina Usenik

**Affiliations:** 1 Department of Agronomy, Chair for Fruit, Viticulture and Vegetable Growing, Biotechnical Faculty, University of Ljubljana, Jamnikarjeva 101, SI-1000 Ljubljana, Slovenia; davor.mrzlic@go.kgzs.si (D.M.); metka.hudina@bf.uni-lj.si (M.H.); valentina.usenik@bf.uni-lj.si (V.U.); 2 Agricultural and Forestry Chamber of Slovenia, Institute of Agriculture and Forestry Nova Gorica, Fruit Growing Centre Bilje, Bilje 1, SI-5292 Rence, Slovenia

**Keywords:** bud, fruit, irrigation, leaf, organic acids, phenolics, *Prunus avium*, sugars

## Abstract

The influence of a water supply on the content of phytochemicals (sugars, organic acids, hydroxycinnamic acids, flavonols, flavanols and anthocyanins) in the bud, leaf and fruit of sweet cherry (*Prunus avium* L.) was studied in two growing seasons. In addition, the shoot length, yield efficiency and fruit weight were determined. The trees of the cultivar ‘Regina’ on Weiroot 72 or Gisela 5 rootstocks were either irrigated or non-irrigated. Irrigated trees received, in addition to rainfall, an amount of water equal to 100% of evapotranspiration, while non-irrigated trees received only rainwater (40% less). An analysis of phytochemicals was performed using high-performance liquid chromatography combined with electrospray ionization mass spectrometry. Irrigated trees had a higher content of total sugars in leaf and bud, higher content of total organic acids in the fruit, and lower content of total hydroxycinnamic acids, total flavonols and flavanols in the leaf and fruit. Irrigated trees also had higher shoot length, fruit weight and lower yield efficiency. The content of phytochemicals in bud and leaf was not affected by rootstock, but the fruit phytochemical composition, shoot length and yield efficiency were. The content of phytochemicals in the bud and leaf was influenced by the presence or absence of fruits. Our results show that irrigation, rootstock and the presence of fruits had an influence on the composition of phytochemicals in sweet cherry.

## 1. Introduction

Water is an important factor in fruit production and irrigation is essential in areas where rainfall is limited or not well distributed. Water supply influences growth [1,2,3,4,5], yield and fruit quality characteristics of sweet cherry (*Prunus avium* L.) [3,6]. The sweet cherry is an economically important stone fruit, which is highly appreciated by consumers. Cherry growers lack scientifically sound information to guide irrigation practices. Adequate water supply is important during flowering and fruit development stages as well as during the postharvest period [7]. The rootstock plays a key role in the tree’s response to water supply [1,8,9,10] and influences numerous scion parameters such as vegetative growth, fruit quality and yield [6], water and gas exchange status [11,12].

Disturbances in the water supply can cause a suppression of photosynthesis, leading to growth-related changes in important biochemical processes in plants [13]. Adaptive responses include accumulation of osmolytes, changes in the composition of metabolites to scavenge reactive oxygen species (ROS) and protection of cell membrane oxidation [14]. Reduced water supply, in general, could lead to altered levels of phytochemicals such as sugars, organic acids and phenolic compounds in plant organs [10,15,16].

Under stress, sugars play an important role in carbon storage by acting as signaling molecules that modulate gene expression and partly scavenge ROS [17]. In response to different water deficits, leaf sugar content increased in *Prunus avium* × *pseudocerasus* ‘Colt’, *Prunus cerasus* ‘Meteor’ [15], peach rootstocks [9], apple [16] and pomegranate [18].

One of the primary changes in response to water deficit may be the formation of ROS, leading to the accumulation of non-enzymatic antioxidant scavengers such as phenolics [19] to counteract the negative effects of stress. Stress can induce phenylpropanoid metabolism and activate enzymes of flavonoid biosynthesis, especially the enzyme phenylalanine ammonia lyase (PAL) [14]. In general, water deficit promotes the accumulation of phenolic compounds [20], such as higher total phenolic content in the leaf of apple ‘Vista Bella’/M9 and pear ‘Santa Maria’/MA [21].

Many research papers have studied the response of sweet cherry to irrigation [1,2,3,4,5,15,22]. Nevertheless, to our knowledge, it is still not known how irrigation alters the content of phytochemicals in the different organs of sweet cherry. Therefore, the aim of this study was to answer the question of whether the response of sweet cherry to irrigation is similar in the sweet cherry’s bud, leaf and fruit.

## 2. Results

### 2.1. Sugars and Organic Acids

**Figure 1:**
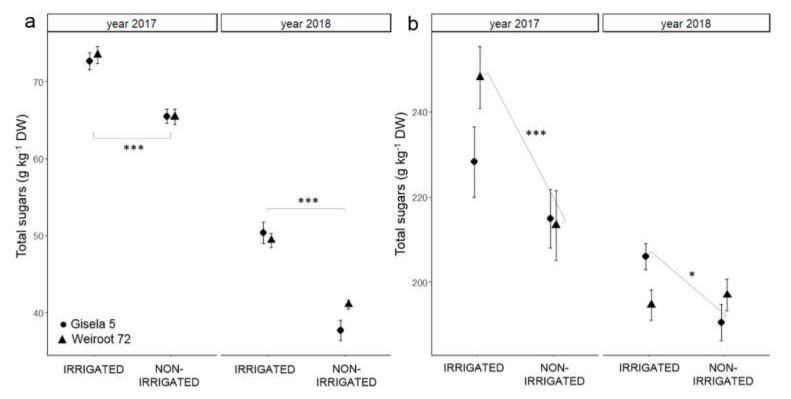
The average content of total sugars (g kg^−1^ DW) in bud (**a**) and leaf (**b**) of irrigated and non-irrigated “Regina” trees on Gisela 5 and Weiroot 72 in 2017 and 2018. Vertical bars represent ± SE of the mean (*n* = 9). Statistically significant differences with respect to irrigation are labeled (*, *p* < 0.05; ***, *p* < 0.001) and connected with the line.

The sugars identified in the bud, leaf and fruit of the sweet cherry were glucose, fructose, sucrose and sorbitol. Sorbitol made up the largest part of the total sugar content measured in the sweet cherry bud and leaf, while glucose accounted for most of the total sugar content in the fruit. The results expressed as average total sugars are shown in Figure 1 (bud and leaf) and in Table 1 (fruit). The highest average total sugar content was measured in fruit (from 725 to 810 g kg^−1^ DW), followed by leaf (from 190 to 248 g kg^−1^ DW) and bud (from 37 to 73 g kg^−1^ DW). In 2017, the average total sugar content was higher in bud and leaf than in 2018 (Figure 1, Tables S1 and S2). The higher average total sugar content was measured in the bud of irrigated trees (Table S5), while the higher average total sugar content in leaf was measured only for irrigated Weiroot 72 in 2017 and Gisela 5 in 2018 (Table S6). 

Irrigation did not affect the average total sugar content in the fruit. Fruit from trees on Gisela 5 had lower average total sugar content than fruit from trees on Weiroot 72 (Table 1). 

The organic acids determined in the fruit were citric acid, malic acid, shikimic acid and fumaric acid. Malic acid was the predominant organic acid. The results for the average total organic acids and sugar/acid ratio in fruits are presented in Table 1. Fruits from irrigated trees had higher average total organic acid content than fruits from non-irrigated trees, while there was no difference in the sugar/acid ratio (Table 1). Lower average total organic acid content was measured in fruits from Gisela 5 than from Weiroot 72 (*p* < 0.01; Table 1). 

### 2.2. Phenolics

Two groups of phenolics were determined in the bud and leaf (hydroxycinnamic acids, flavonols and flavanols), while three groups of phenolics were determined in the fruit (hydroxycinnamic acids, flavonols and flavanols, anthocyanins).

**Figure 2:**
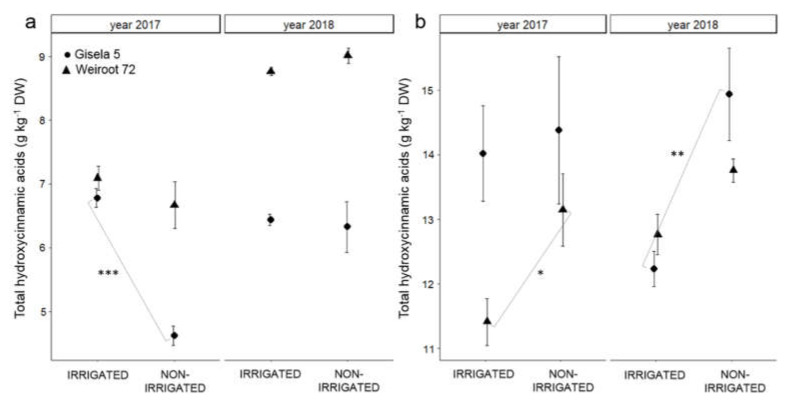
The average content of total hydroxycinnamic acids (g kg^−1^ DW) in bud (**a**) and leaf (**b**) of irrigated and non-irrigated ‘Regina’ trees on Gisela 5 and Weiroot 72 in 2017 and 2018. Vertical bars represent ± SE of the mean (*n* = 9). Statistically significant differences with respect to irrigation are labeled (*, *p* < 0.05; **, *p* < 0.01; ***, *p* < 0.001) and connected with the line.

The hydroxycinnamic acids identified in the bud were neochlorogenic acid, chlorogenic acid, caffeoylquinic acid and *p*-coumaroylquinic acid. In the leaf, the hydroxycinnamic acids identified were neochlorogenic acid, chlorogenic acid, caffeoylquinic acid, 3,5-di-*O*-caffeoylquinic acid, 3,4-di-*O*-caffeoylquinic acid, and *p*-coumaroylquinic acid. In the fruit, they were neochlorogenic acid, dicaffeoylquinic acid and *p*-coumaroylquinic acid. The highest average total hydroxycinnamic acid content was measured in the leaf (from 11.1 to 14.4 g kg^−1^ DW), followed by the bud (from 4.6 to 9.0 g kg^−1^ DW) and the fruit (from 1.0 to 1.2 g kg^−1^ DW). The bud of irrigated trees had similar average total hydroxycinnamic acid content to that of non-irrigated trees, except for Gisela 5 in 2017 (non-irrigated less than irrigated; Figure 2 and Table S5). In the leaf, irrigation influenced lower total hydroxycinnamic acid content, with a statistically significant difference for Weiroot 72 in 2017 and Gisela 5 in 2018 (Figure 2 and Table S6). 

Fruit from irrigated trees of Weiroot 72 had lower average total hydroxycinnamic acid content than fruit from non-irrigated trees, while no differences were observed for fruit of Gisela 5 (Table 1). 

**Figure 3:**
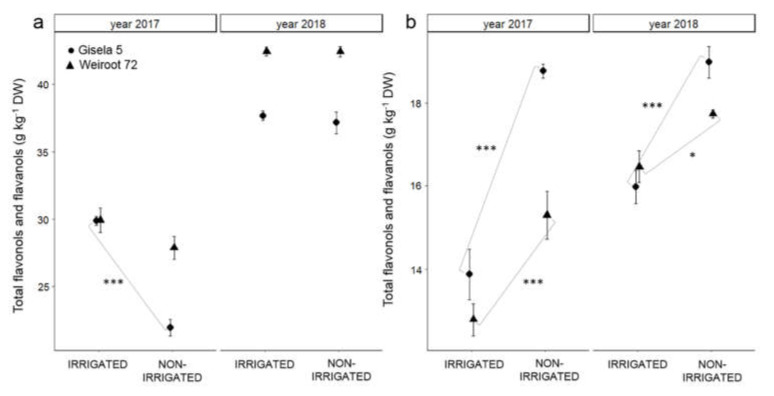
The average content of total flavonols and flavanols (g kg^−1^ DW) in bud (**a**) and leaf (**b**) of irrigated and non-irrigated ‘Regina’ trees on Gisela 5 and Weiroot 72 in 2017 and 2018. Vertical bars represent ± SE of the mean (*n* = 9). Statistically significant differences with respect to irrigation are labeled (*, *p* < 0.05; ***, *p* < 0.001) and connected with the line.

The flavonols and flavanols identified in the bud were quercetin-3-*O*-rutinoside, quercetin-3-*O*-glucoside, quercetin-diglucoside, kaempferol-3-*O*-rutinoside, catechin, epicatechin, procyanidin B1, procyanidin B2, procyanidin trimer. In the leaf, the flavonols and flavanols identified were quercetin-3-*O*-rutinoside, quercetin-3-*O*-glucoside, quercetin-diglucoside, kaempferol-3-*O*-glucoside, catechin and epicatechin. In the fruit, they were quercetin-3-(*2G*)-glucosylrutinoside, quercetin-3-*O*-rutinoside, quercetin-3-*O*-glucoside, kaempferol-3-*O*-rutinoside, epicatechin and procyanidin B2. The bud showed the highest average total flavonols and flavanols (from 22.0 to 42.4 g kg^−1^ DW), followed by the leaf (from 12.8 to 19.0 g kg^−1^ DW) and fruit (from 0.9 to 1.1 g kg^−1^ DW). In 2018, the average content of total flavonols and flavanols in the bud and leaf was higher than in 2017 (Figure 3, Tables S5 and S6). Irrigated trees had similar average content of total flavonols and flavanols in the bud as non-irrigated trees. The only exception was Gisela 5 in 2017, where bud from irrigated trees had higher average total flavonol and flavanol content than bud from non-irrigated trees. Irrigation influenced the lower content of total flavonols and flavanols in leaf and fruit (*p* < 0.001 and *p* < 0.01, respectively; Figure 3 and Table 1). 

The anthocyanins identified in sweet cherry fruit were cyanidin-3-*O*-glucoside, cyanidin-3-*O*-rutionoside, pelargonidin-3-*O*-rutinoside and peonidin-3-*O*-rutinoside. Cyanidin-3-*O*-rutionoside made up the largest part of total anthocyanins. The results, expressed as total anthocyanins, are shown in Table 1. The average content of total anthocyanins in fruits ranged from 2.6 to 3.1 g kg^−1^ DW. Irrigation had no effect on the average total anthocyanin content. The ANOVA showed a statistically significant influence of rootstock (*p* < 0.001). The fruit from trees on Gisela 5 had lower average total anthocyanin content than fruit from trees on Weiroot 72 (Table 1). 

### 2.3. Shoot Length, Yield Efficiency and Fruit Weight Measurements

**Figure 4:**
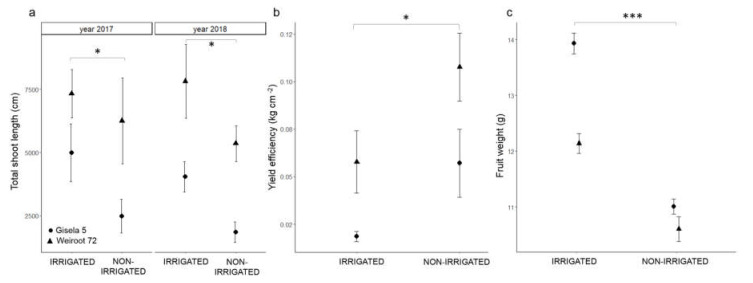
The average total shoot length in years 2017 and 2018 (**a**), yield efficiency (**b**) and average fruit weight (**c**) in 2018 of irrigated and non-irrigated ‘Regina’ trees on Gisela 5 and Weiroot 72. Vertical bars represent ± SE of the mean (*n* = 3 for total shoot length and yield efficiency and *n* = 20 for fruit weight). Statistically significant differences with respect to irrigation are labeled (*, *p* < 0.05; ***, *p* < 0.001) and connected with the line.

Irrigation and rootstock influenced total shoot length and yield efficiency (Tables S3 and S4). Irrigated trees had a higher average total shoot length, higher fruit weight and lower yield efficiency than non-irrigated trees (Figure 4 and Table S7). Total shoot length and yield efficiency were higher on Weiroot 72 than on Gisela 5 (Figure 4 and Table S7). 

## 3. Discussion

The effects of irrigation on sweet cherry trees were studied by analyzing phytochemicals in bud, leaf and fruit, and by measuring shoot length, yield efficiency and fruit weight in two successive growing seasons. Measurements of phytochemical content in leaf and bud were made to obtain indirect information on assimilation rates in the leaf. Individual sugars were previously determined in the leaf of *Prunus avium* × *pseudocerasus* and *Prunus cerasus* [15], the bud of *P. avium* [23,24] and fruit of *P. avium* [25,26]. The organic acids identified in our study are the same as those previously identified in the fruit of *P. avium* [25]. The phenolic compounds identified in the bud, leaf and fruit of sweet cherry are the same as those previously identified in the bud of *P. avium* [24,27], leaf of *P. cerasus* [28] and fruit of *P. avium* [25,29].

In 2017, the total sugar content in bud and leaf was higher than in 2018, when the trees were fruiting. The lower total sugar content in bud and leaf of trees bearing fruits shows the influence of sink on phytochemical composition. When the main sink is absent, sugar is transported to other sinks, and the sugar content increases in the organs, as in the leaf or bud in our study. It was previously reported that the content of sugars in the leaf is regulated by the balance between synthesis, degradation and export [16]. Our results also show the correlation between leaf and bud sugar content. In general, high sugar content in the leaf led to high sugar content in the bud (in 2017), and low content in the leaf led to low content in the bud (in 2018). Lower total sugar content was measured in the leaf of non-irrigated trees on Weiroot 72 in 2017 and on Gisela 5 in 2018. This may be related to photosynthetic disorders, which are considered as the main cause of inhibition of photosynthesis and thus impaired carbohydrate production [13,18]. Ebtedaie and Shekafandeh [18] found no differences between control trees and trees receiving 25% or 75% less water. Non-irrigated trees had lower total sugar content in buds than irrigated trees. Sugars are the major assimilates provided by leaves through processes of photosynthesis [24]. The lower sugar content in the leaves of non-irrigated trees is associated with lower net photosynthesis during the growing season, which is reflected in lower storage of reserves in the buds such as sugars. It was previously reported that exposure to abiotic stresses, such as water deficit, is related to intra-plant competition for carbon and may additionally lower sugar level within the plant [30].

Irrigation had no effect on the average total sugar content of the fruit in our study, but it increased the organic acid content. Sugars, organic acids and the ratio between them (sugar/acid ratio) are an important component in the flavor formation of fruits [31]. We can assume that fruits from irrigated trees were tastier than those from non-irrigated trees due to higher organic acid content, although the difference between irrigated and non-irrigated trees in the sugar/acid ratio was not significant. The similar sugar content in fruit from irrigated and non-irrigated trees could be due to the same degree of ripeness of the fruits used for biochemical analysis. Since the maturity level of fruits was not unique, and although the optimum maturity for the ‘Regina’ fruit is CTIFL 5-6, only CTIFL 4 fruits were used for biochemical analysis to reduce the influence of maturity. Fruit color correlates with the degree of ripeness of sweet cherry fruit [32], and among other factors, determines the fruit sugar content [33]. Another reason for the lack of differences in total sugar content may be the extent of water deficit of the non-irrigated trees in our study, which was probably not high enough to cause osmoprotection [34]. When plants are subjected to moderate water stress, an osmotic adjustment may occur without osmoprotection but by enhancing the plant’s ability to absorb water [35]. Our results confirm the findings of Demirtas et al. [3], who found no differences in sugar content of sweet cherry fruit of the same maturity under different water supply treatments. 

In general, the non-irrigated trees showed higher contents of total hydroxycinnamic acids, total flavonols and flavanols in the leaf and fruit. The results suggest that the non-irrigated trees in our study may have suffered from water deficiency, which was reflected in the accumulation of antioxidant compounds such as hydroxycinnamic acids, flavonols and flavanols. In agreement with our results, Bolat et al. [21] reported higher total phenolic content in the leaf of apple and pear trees exposed to water deficit. The reduced water supply could increase the production of ROS, and oxidative stress may occur, which is known to be one of the major causes of plant damage [19]. As a result, various antioxidants such as secondary metabolites, especially flavonoids and hydroxycinnamic acids, accumulate in plant tissues and provide antioxidant protection against ROS, before damaging the cell [19]. Moreover, a reduced water supply was shown to induce phenol accumulation by up-regulating and inducing gene expression and enzyme activity of PAL [36]. Moreover, total flavonol and flavanol content of bud and leaf were higher on bearing trees, indicating altered plant metabolism due to the presence of fruits. An even higher total flavonol and flavanol content in the leaf of non-irrigated trees indicated a higher stress level, which was probably related to the water supply.

The water supply did not affect the total anthocyanin content of sweet cherry fruit. This result confirms the same ripening stage of the fruits used for the biochemical analyses. Phenolic content is strongly related to the ripening stage, with riper fruit containing more phenolics [32]. Total anthocyanin and total hydroxycinnamic acid contents were higher on Weiroot 72, indicating a better assimilative capacity of the tree on a more vigorous rootstock. The influence of water supply on the synthesis of phytochemicals by the rootstock in *P. persica* was reported previously [10]. Irrigated trees exhibited an increased shoot length irrespective of rootstock. Our results are in agreement with other reports [1,4,5] that vegetative growth increases with irrigation and with more vigorous rootstocks. The reduced shoot length of non-irrigated trees could be a consequence of reduced CO_2_ fixation as a result of closed stomata [37]. It is interesting to note that the shoot length of bearing trees in 2018 was similar to that of non-bearing trees in 2017. This phenomenon may be explained by the sugar content in the bud. Even though the biochemical analyses were performed on the reproductive bud, we can assume that the content in the vegetative bud would be similar. The trees accumulated more sugars in the non-bearing year, leading to vigorous growth in the following year, which was not diminished by the presence of fruit. We can therefore assume that low sugar content in the bud is the basis for poor vegetative growth in the following fruit-bearing year.

Yield efficiency, a common parameter when the size of the trees used in the experiment is not equal, indirectly indicates the number of fruits per tree. The higher number of fruits from non-irrigated trees in our study was probably stimulated by the lower vegetative growth, but the lower fruit weight was a consequence of the low leaf/fruit ratio [38]. Our results are in agreement with those of Razouk et al. [39]. In contrast, Podestá et al. [5] and Demirtas et al. [3] showed no differences in the fruit weight of ‘Bing’ and ‘0900-Ziraat’ on vigorous rootstocks MaxMa 14 and Mazzard, respectively, under irrigation treatments. Fruit growth was reported to be generally less sensitive to water scarcity than vegetative growth [40]. 

## 4. Materials and Methods

### 4.1. Experimental Design

The experiment was conducted on sweet cherry (*Prunus avium* L.) at the Fruit Growing Center Bilje in Slovenia (45° 53′ latitude, 13° 38′ longitude, 55 m altitude). Trees of the cultivar ‘Regina’ on Gisela 5 or Weiroot 72 (dwarf or semi-dwarf rootstock) at a planting distance of 4 × 2.5 m (Weiroot 72) or 4 × 2 m (Gisela 5) were irrigated or non-irrigated (control) over the years. Non-irrigated trees received rainwater only, but irrigated trees received additional water through under-tree-micro-irrigation to cover 100% of the reference evapotranspiration (ETo) from May to August. Non-irrigated trees received 40% less water than irrigated trees. Plant water requirements were estimated from plant evapotranspiration (ETc), calculated using the equation Penman–Monteith (ETc = ETo × Kc) [41]. The crop coefficient (Kc) was estimated using FAO Irrigation and Drainage Paper [41], adjusted for the agricultural and environmental conditions of the experimental site and plant phenological stage. Three of five trees per rootstock and irrigation treatment were included in the sampling and measurements in 2017 and 2018, while avoiding marginal trees. Environmental conditions are shown in Supplementary Figure S1.

### 4.2. Sampling and Sample Preparation for Biochemical Analysis

Thirty dormant reproductive buds per tree were sampled from spurs during the dormant period in December. Fifteen fully developed and undamaged leaves per tree of similar exposure were sampled in July from the middle of the current season shoots. Twenty fruits per tree of quality class CTIFL 4 were sampled in 2018 on 15 June. The harvest could only be assessed in 2018, while in 2017 the spring frost destroyed the fruit. Each bud and leaf sample was divided into three subsamples, but each fruit sample was divided into four subsamples. The plant material was immediately frozen in liquid nitrogen, lyophilized, ground to a fine powder in a cooled grinder and stored in moisture-proof, dark, plastic containers at −20 °C until analysis. 

### 4.3. Sugars and Organic Acids

The extraction of sugars and organic acids was carried out separately as follows: 0.5 g of bud, 0.4 g of leaf or 0.3 g of fruit were homogenized in 5 mL (bud and fruit) or 7 mL (leaf) of double distilled water and left for 30 min at room temperature with frequent stirring. The extracts were centrifuged at 11,500× *g* for 10 min at 4 °C (Eppendorf Centrifuge 5810R, Hamburg, Germany). After extraction, the supernatants were filtered (0.20 μm, Chromafil A-20/25, Macherey-Nagel, Düren, Germany) and transferred to vials [25]. 

The analysis of sugars and organic acids was performed according to Usenik et al. [25] on a Surveyor high-performance liquid chromatography system (Thermo Scientific, Finnigan Spectra system, Waltham, MA, USA) with a refractive index detector (Thermo Finnigan, San Jose, CA, USA; sugars) or UV detector (organic acids). A Rezex-RCM-monosaccharide Ca^2+^ (2%) column (300 mm × 7.8 mm; Phenomenex, Torrance, CA, USA) was used for the analysis of sugars, and a Rezex-ROA column (300 mm × 7.8 mm; Phenomenex, Torrance, CA, USA) operated at 65 °C was used for organic acids. The elution solvent was double distilled water for sugars and 4 mM sulfuric(VI) acid for organic acids. The injection volume was 20 μL, the flow rate was 0.6 mL min^−1^, and the run time was 30 min. The quantification of the identified compounds was evaluated from peak areas, calculated from a calibration curve of the corresponding standard and expressed in grams per kilogram dry weight (g kg^−1^ DW).

### 4.4. Phenolics

Extraction of phenolics was performed as follows: 0.08 g of bud, 0.04 g of leaf, or 0.4 g of fruit were homogenized in 10 ml 80% methanol containing 3% formic acid (*v*/*v*) by vortexing and extracted in a cooled ultrasonic bath for 1 h. After extraction, samples were centrifuged at 11,500× *g* and 4 °C for 10 min (Eppendorf Centrifuge 5810R, Hamburg, Germany), filtered through 0.2 µm polyamide filters (Chromafil AO-20/25, Macherey-Nagel, Düren, Germany) and transferred into vials [25].

The analysis of phenolic compounds was performed on the Dionex UltiMate 3000 HPLC system (Thermo Scientific, San Jose, CA, USA), monitoring absorbance at 280 nm (hydroxycinnamic acids), 350 nm (flavonols and flavanols) and 530 nm (anthocyanins). Separation was performed on a Gemini C_18_ (150 mm × 4.6 mm, 3 µm, Phenomenex, Torrance, CA, USA) column at 25 °C. Mobile phase A consisted of 3% acetonitrile with 0.1% formic acid in double-distilled water (*v/v/v*), and mobile phase B consisted of 3% double-distilled water with 0.1% formic acid in acetonitrile (*v/v/v*). The following linear gradient was used: 0–15 min, 5% solvent B; 15–20 min, 20% B; 20–30 min, 30% B; 30–35 min, 90% B; and 35–45 min, 100% B before returning to initial conditions until the end of the run (50 min). The injection volume was 20 µL and the flow rate was 0.6 ml min^−1^ [25].

Identification of phenolic compounds was confirmed by spectral characteristics, comparison of retention times, and use of the LCQ Deca XP MAX mass spectrometer (Thermo Finnigan, San Jose, CA, USA) with electrospray ionization (ESI) operating in negative ion mode. The content of each compound was calculated from a calibration curve of the corresponding standard and expressed in g kg^−1^ DW. 

Quantification of compounds without standards was performed with similar compounds; dicaffeoylquinic acid, 3,5-di-*O*-caffeoylquinic acid, 3,4-di-*O*-caffeoylquinic acid and caffeoyllquinic acid as the equivalent of chlorogenic acid; quercetin-diglucoside as the equivalent of quercetin-3-*O*-glucoside; *p*-coumaroylquinic acid as the equivalent of *p*-coumaric acid; quercetin-3-(*2G*)-glucosylrutinoside as the equivalent of quercetin-3-*O*-galactoside; kaempferol-3-*O*-rutinoside as the equivalent of kaempferol-3-*O*-glucoside; pelargonidin-3-*O*-rutinoside as the equivalent of pelargonidin-3-*O*-glucoside and peonidin-3-*O*-rutinoside as the equivalent of peonidin-3-*O*-glucoside.

The sum of all identified sugars, organic acids, hydroxycinnamic acids, flavonols, flavanols and anthocyanins was calculated and presented as total sugars, total organic acids, total hydroxycinnamic acids, total flavonols and flavanols and total anthocyanins. In addition, the sugar/acid ratio (ratio between total sugars and total organic acids) was calculated. 

### 4.5. Chemicals

The following standards were used to identify and quantify compounds: glucose, fructose, sucrose, sorbitol, citric acid, malic acid, fumaric acid and shikimic acid, quercetin-3-*O*-galactoside, quercetin- and kaempferol-3-*O*-glucoside, (–)-epicatechin, (+)-catechin, *p*-coumaric acid, procyanidin B2, cyanidin-3-*O*-rutinoside (keracyanin), pelargonidin-3-*O*-glucoside and peonidin-3-*O*-glucoside from Fluka Chemie GmbH (Buchs, Switzerland); neochlorogenic (3-caffeoylquinic) acid, chlorogenic (5-caffeoylquinic) acid, quercetin-3-*O*-rutinoside (rutin) and cyanidin-3-*O*-glucoside from Sigma–Aldrich Chemical (St. Louis, MO, USA). Methanol for the extraction of phenolics was obtained from Sigma–Aldrich Chemical (St. Louis, MO, USA). The chemicals for the mobile phases were HPLC–MS grade acetonitrile and formic acid purchased from Fluka Chemie GmbH (Buchs, Switzerland). The water for the mobile phase was double distilled and purified using the Milli-Q system (Millipore, Bedford, MA, USA). 

### 4.6. Shoot Length, Yield Efficiency and Fruit Weight Measurements

At the end of growing seasons in 2017 and 2018, the trunk diameter and length of the current year’s shoots were measured, and total shoot length was calculated as the sum of the length of all shoots per tree. The harvest could only be assessed in 2018, while in 2017 the spring frost destroyed the fruit. Fruits were picked all at once on 15 June and classified into quality classes using the CTIFL chart (Centre Technique Interprofessionnel des Fruits et Légumes; Paris, France). Twenty fruit per tree were taken from fruit classified as CTIFL 4 for measurements of fruit weight. The yield efficiency was calculated as the ratio between yield and trunk cross-sectional area calculated from trunk diameter and expressed in kg cm^-2^.

### 4.7. Statistical Analysis

A statistical analysis was performed using R statistical environment version 3.6.1 [42]. The leaf and bud phytochemicals and shoot length data were subjected to a three-factor analysis of variance (ANOVA, factors year, rootstock, and irrigation), while the data of fruit phytochemicals, yield efficiency and fruit weight were subjected to a two-factor ANOVA (rootstock and irrigation). The year factor had two levels (2017 and 2018), the rootstock factor had two levels (Gisela 5 and Weiroot 72) and the irrigation factor had two levels (irrigated and non-irrigated). When the ANOVA showed statistical significance (Supplementary Tables S1–S4), a contrast analysis was performed using the *glht* function with user-defined contrasts (Supplementary Tables S5–S7) from the R package *multcomp* with a generalized hypothesis testing procedure, considering simultaneous hypothesis tests. If the *p* value for differences between means was less than 0.05, the difference was considered statistically significant. 

## 5. Conclusions

In conclusion, the present study showed the influence of irrigation on the content of phytochemicals (sugars, organic acids and phenolics) in bud, leaf and fruit and on shoot length, yield efficiency and fruit weight of sweet cherry on two rootstocks in two growing seasons. Phytochemical composition differed among the organs observed. Irrigated trees had higher total sugar content in bud and leaf, lower total hydroxycinnamic acid, total flavonol and flavanol content in leaf, higher total shoot length, lower yield efficiency and higher fruit weight, with some exceptions. Fruit from irrigated trees showed higher total organic acid content and lower total hydroxycinnamic acid and total flavonol and flavanol content. Water deficiency was reflected in higher phenolic contents in the leaf and fruit. Rootstock affected total shoot length, yield efficiency, total sugar, total organic acid, and total anthocyanin content in fruit, while the presence or absence of fruit affected total sugar, and total flavonol and flavanol content in the bud and leaf. Our results indicate that irrigation, rootstock and the presence of fruit determine the composition of phytochemicals in sweet cherry organs.

## Figures and Tables

**Table 1:** The average (± SE) content of total sugars, total organic acids, total hydroxycinnamic acids, total flavonols and flavanols, total anthocyanins (g kg^−1^ DW) and sugar/acid ratio in fruit from irrigated and non-irrigated ‘Regina’ trees on Gisela 5 and Weiroot 72 in 2018 (*n* = 12).

	Irrigated		Non-irrigated		ANOVA ^a^		
	Gisela 5	Weiroot 72	Gisela 5	Weiroot 72	I	R	I × R
Total sugars	740.3 ± 19.8 Y	810.4 ± 21.8 X	724.5 ± 17.4 Y	765.5 ± 25.8 X	*NS*	***	*NS*
Total organic acids	70.9 ± 3.4 AY	78.0 ± 2.2 AX	62.7 ± 1.7 BY	68.5 ± 0.9 BX	***	**	*NS*
Total hydroxycinnamic acids	1.01 ± 0.02 ab	1.10 ± 0.02 a	1.01 ± 0.03 ab	1.22 ± 0.04 b	*	***	*
Total flavonols and flavanols	0.95 ± 0.04 B	0.92 ± 0.03 B	1.01 ± 0.03 A	1.07 ± 0.04 A	**	*NS*	*NS*
Total anthocyanins	2.6 ± 0.1 Y	3.1 ± 0.1 X	2.5 ± 0.1 Y	3.1 ± 0.1 X	*NS*	***	*NS*
Sugar/acid ratio	10.4 ± 0.7	10.3 ± 0.4	11.6 ± 0.4	11.2 ± 0.4	*NS*	*NS*	*NS*

Mean values followed by different uppercase letters in a row indicate statistically significant differences between irrigation treatments (AB) or between rootstocks (XY; contrast analysis, *p* < 0.05); mean values followed by different lowercase letters in a row indicate statistically significant differences for the interaction (contrast analysis, *p* < 0.05). ^a^– I, irrigation; R, rootstock; I × R, interaction; *, statistically significant differences at *p* < 0.05; **, statistically significant differences at *p* < 0.01; ***, statistically significant differences at p < 0.001; NS, not significant.

## Supplementary Materials

The following are available online at https://www.mdpi.com/article/10.3390/plants10061131/s1, Figure S1: Average daily air temperature (°C), precipitation (mm) and reference evapotranspiration (ETo, mm) from May to August in 2017 and 2018 in Fruit Growing Center Bilje, Slovenia, Table S1: Statistically significant differences (ANOVA) for main effects and interactions for the content of total sugars (TS), total flavonols and flavanols (TFF) and total hydroxycinnamic acids (THCA) in bud of ‘Regina’ on Weiroot 72 or Gisela 5, Table S2: Statistically significant differences (ANOVA) for main effects and interactions for the content of total sugars (TS), total flavonols and flavanols (TFF) and total hydroxycinnamic acids (THCA) in leaf of ‘Regina’ on Weiroot 72 or Gisela 5, Table S3: Statistically significant differences (ANOVA) for main effects and interactions for total shoot length (TSL) of sweet cherry ‘Regina’ on Weiroot 72 or Gisela 5, Table S4: Statistically significant differences (ANOVA) for main effects and interactions for the measurements of yield efficiency (YE) and fruit weight (FW) of sweet cherry ‘Regina’ on Weiroot 72 or Gisela 5, Table S5: Contrast analysis results: statistically significance of selected comparisons depending on the analysis of variance for main effects and/or interactions for the content of total sugars (TS), total flavonols and flavanols (TFF) and total hydroxycinnamic acids (THCA) in bud of ‘Regina’ on Weiroot 72 or Gisela 5, Table S6: Contrast analysis results: statistically significance of selected comparisons depending on the analysis of variance for interaction for the content of total sugars (TS), total flavonols and flavanols (TFF) and total hydroxycinnamic acids (THCA) in leaf of ‘Regina’ on Weiroot 72 or Gisela 5, Table S7: Contrast analysis results: statistically significance of selected comparisons depending on the analysis of variance for main effects for the total shoot length (TSL), yield efficiency (YE) and fruit weight (FW) of ‘Regina’ on Weiroot 72 or Gisela 5.
