# Supplementary material for: The Effect of Water Supply on Sweet Cherry Phytochemicals in Bud, Leaf and Fruit
Source: Plants (Basel). 2021 Jun 2;10(6):1131. doi: 10.3390/plants10061131 (PMC8228343; doi:10.3390/plants10061131)
Supplement: Supplementary file 1 [file plants-10-01131-s001.zip › plants-1240764-supplementary.pdf]

SUPPLEMENTARY MATERIAL

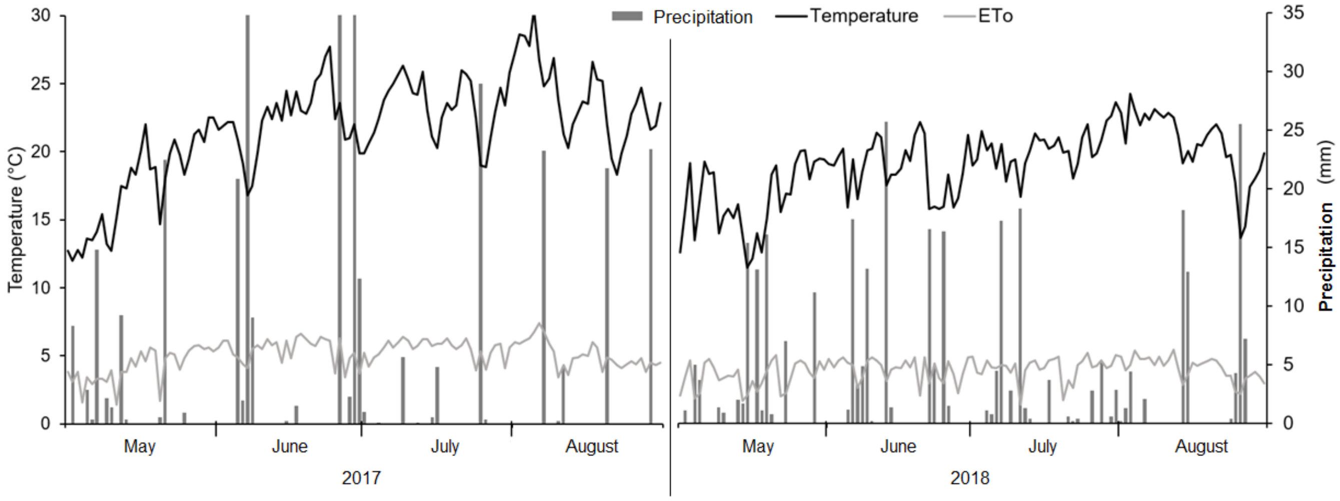

Figure S1: Average daily air temperature (°C), precipitation (mm) and reference evapotranspiration (ETo, mm) from May to August in 2017 and 2018 in Fruit Growing Center Bilje, Slovenia.

Table S1: Statistically significant differences (ANOVA) for main effects and interactions for the content of total sugars (TS), total flavonols and flavanols (TFF) and total hydroxycinnamic acids (THCA) in bud of 'Regina' on Weiroot 72 or Gisela 5.

Main effect/interaction[a]	df	TS	TFF	THCA
I	1	***	***	***
R	1	*NS*	***	***
Y	1	***	***	***
I×R	1	*NS*	**	**
I×Y	1	*NS*	***	***
R×Y	1	*NS*	*NS*	***
I×R×Y	1	*NS*	*	*

[a]ANOVA – I, irrigation; R, rootstock, Y, year; ×, interaction; *, statistically significant differences at $p < 0.05$; **, statistically significant differences at $p < 0.01$; ***, statistically significant differences at $p < 0.001$; *NS*, not significant; df, degrees of freedom.

Table S2: Statistically significant differences (ANOVA) for main effects and interactions for the content of total sugars (TS), total flavonols and flavanols (TFF) and total hydroxycinnamic acids (THCA) in leaf of 'Regina' on Weiroot 72 or Gisela 5.

Main effect/interaction[a]	df	TS	TFF	THCA
I	1	***	***	**
R	1	*NS*	***	*
Y	1	***	***	*NS*
I×R	1	*NS*	***	*NS*
I×Y	1	*	**	*NS*
R×Y	1	*NS*	**	*
I×R×Y	1	*	*	*

[a]ANOVA – I, irrigation; R, rootstock, Y, year; ×, interaction; *, statistically significant differences at $p < 0.05$; **, statistically significant differences at $p < 0.01$; ***, statistically significant differences at $p < 0.001$; *NS*, not significant; df, degrees of freedom.

Table S3: Statistically significant differences (ANOVA) for main effects and interactions for the measurements of total shoot length (TSL) of sweet cherry 'Regina' on Weiroot 72 or Gisela 5.

Main effect/interaction[a]	df	TSL
I	1	*
R	1	***
Y	1	*NS*
I×R	1	*NS*
I×Y	1	*NS*
R×Y	1	*NS*
I×R×Y	1	*NS*

[a]ANOVA – I, irrigation; R, rootstock, Y, year; ×, interaction; *, statistically significant differences at $p < 0.05$; **, statistically significant differences at $p < 0.01$; ***, statistically significant differences at $p < 0.001$; *NS*, not significant; df, degrees of freedom.

Table S4: Statistically significant differences (ANOVA) for main effects and interactions for the measurements of yield efficiency (YE) and fruit weight (FW) of sweet cherry 'Regina' on Weiroot 72 or Gisela 5.

Main effect/interaction[a]	df	YE	FW
I	1	*	**
R	1	*	*NS*
I×R	1	*NS*	*NS*

[a]ANOVA – I, irrigation; R, rootstock; ×, interaction; *, statistically significant differences at $p < 0.05$; **, statistically significant differences at $p < 0.01$; ***, statistically significant differences at $p < 0.001$; *NS*, not significant; df, degrees of freedom.

Table S5: Contrast analysis results: statistically significance of selected comparisons depending on the analysis of variance for main effects and/or interactions for the content of total sugars (TS), total flavonols and flavanols (TFF) and total hydroxycinnamic acids (THCA) in bud of 'Regina' on Weiroot 72 or Gisela 5.

Main effect/interaction	Comparison	TS	TFF	THCA
I	Irr. – Non-irr.	↑***	—	—
Y	'17 – '18	↑***	—	—
I×R×Y	Irr.G5.'17 – Non-irr.G5.'17	—	↑***	↑***
	Irr.W72.'17 – Non-irr.W72.'17	—	*NS*	*NS*
	Irr.G5.'18 – Non-irr.G5.'18	—	*NS*	*NS*
	Irr.W72.'18 – Non-irr.W72.'18	—	*NS*	*NS*
	Irr.G5.'17 – Irr.G5.'18	—	↓***	*NS*
	Irr.W72.'17 – Irr.W72.'18	—	↓***	↓***
	Non-irr.G5.'17 – Non-irr.G5.'18	—	↓***	↓***
	Non-irr.W72.'17 – Non-irr.W72.'18	—	↓***	↓***

Statistically significant differences at *** $p < 0.001$; *NS* - not significant; ↑ = increase in the first pair of comparison compared to second; ↓ = decrease in the first pair of comparison compared to second; I, irrigation; R, rootstock, Y, year; ×, interaction; —, not analyzed; Irr., irrigated trees; Non-irr., non-irrigated trees; G5, Gisela 5 rootstock; W72, Weiroot 72 rootstock; '17, year 2017; '18, year 2018.

Table S6: Contrast analysis results: statistically significance of selected comparisons depending on the analysis of variance for interaction for the content of total sugars (TS), total flavonols and flavanols (TFF) and total hydroxycinnamic acids (THCA) in leaf of 'Regina' on Weiroot 72 or Gisela 5.

Interaction	Comparison	TS	TFF	THCA
I×R×Y	Irr.G5.'17 – Non-irr.G5.'17	*NS*	↓***	*NS*
	Irr.W72.'17 – Non-irr.W72.'17	↑***	↓***	↓*
	Irr.G5.'18 – Non-irr.G5.'18	↑*	↓***	↓**
	Irr.W72.'18 – Non-irr.W72.'18	*NS*	↓*	*NS*
	Irr.G5.'17 – Irr.G5.'18	↑*	↓***	↑*
	Irr.W72.'17 – Irr.W72.'18	↑***	↓***	*NS*
	Non-irr.G5.'17 – Non-irr.G5.'18	↑**	*NS*	*NS*
	Non-irr.W72.'17 – Non-irr.W72.'18	↑*	↓***	*NS*

Statistically significant differences at *** $p < 0.001$, ** $p < 0.01$, * $p < 0.05$; *NS* - not significant; ↑ = increase in the first pair of comparison compared to second; ↓ = decrease in the first pair of comparison compared to second; I, irrigation; R, rootstock, Y, year; ×, interaction; Irr., irrigated trees; Non-irr., non-irrigated trees; G5, Gisela 5 rootstock; W72, Weiroot 72 rootstock; '17, year 2017; '18, year 2018.

Table S7: Contrast analysis results: statistically significance of selected comparisons depending on the analysis of variance for main effects for the total shoot length (TSL), yield efficiency (YE) and fruit weight (FW) of 'Regina' on Weiroot 72 or Gisela 5.

Main effect	Comparison	TSL	YE	FW
I	Irr. – Non-irr.	↑*	↓*	↑***
R	G5 – W72	↓**	↓**	—

Statistically significant differences at *** $p < 0.001$, ** $p < 0.01$, * $p < 0.05$; *NS* - not significant; ↑ = increase in the first pair of comparison compared to second; ↓ = decrease in the first pair of comparison compared to second; —, not analyzed; Irr., irrigated trees; Non-irr., non-irrigated trees; G5, Gisela 5 rootstock; W72, Weiroot 72 rootstock.
